# Morphological, Chemical, and Genetic Characteristics of Korean Native Thyme Bak-Ri-Hyang (*Thymus quinquecostatus* Celak.)

**DOI:** 10.3390/antibiotics9060289

**Published:** 2020-05-28

**Authors:** Minju Kim, Jun-Cheol Moon, Songmun Kim, Kandhasamy Sowndhararajan

**Affiliations:** 1 School of Natural Resources and Environmental Science, Kangwon National University, Chuncheon 24341, Gangwon-do, Korea; scent@kangwon.ac.kr; 2 Agriculture and Life Sciences Research Institute, Kangwon National University, Chuncheon 24341, Gangwon-do, Korea; jhmoon73@gmail.com; 3 Department of Botany, Kongunadu Arts and Science College, Coimbatore 641029, Tamil Nadu, India

**Keywords:** essential oil, genetic, RAPD, thyme, *Thymus quinquecostatus*, *Thymus vulgaris*

## Abstract

Bak-ri-hyang (*Thymus quinquecostatus* Celak.) is an important medicinal and aromatic plant in Korea. *T. quinquecostatus* population and is always mixed with other thyme cultivars during cultivation and marketing. Hence, this study aimed to determine the genetic variability and the essential oil composition of three Korean native thyme, *T. quinquecostatus* cultivars collected from the Wolchul, Jiri, and Odae mountains, in comparison with six commercial thyme cultivars (*T. vulgaris*), to distinguish Bak-ri-hyang from other thyme cultivars. The composition of essential oils obtained from nine individuals was analyzed by gas chromatography–mass spectrometry (GC–MS). The random amplified polymorphic DNA (RAPD) analysis was accomplished using 16 different primers. The GC–MS analysis revealed that Wolchul, creeping, golden, and orange cultivars belong to the geraniol chemotype. Whereas the Odae, lemon, and silver cultivars belong to the thymol chemotype. Further, linalool was the most abundant component in carpet and Jiri cultivars. The RAPD analysis demonstrated that all thyme cultivars showed characteristic RAPD patterns that allowed their identification. In total, 133 bands were obtained using 16 primers, and 124 bands were polymorphic, corresponding to 93.2% polymorphism. Cluster analysis of RAPD markers established the presence of clear separation from nine thyme cultivars. The highest dissimilarity and similarity coefficient of the RAPD markers were 0.58 and 0.98, respectively. According to the RAPD patterns, the nine thyme cultivars could be divided into two major clusters. Among three Korean cultivars, the Wolchul and Odae cultivars were placed into the same cluster, but they did not show identical clustering with their essential oil compositions. The findings of the present study suggest that RAPD analysis can be a useful tool for marker-assisted identification of *T. quinquecostatus* from other *Thymus* species.

## 1. Introduction

The genus *Thymus* (Lamiaceae) consists of approximately 300 species of herbaceous perennials and sub-shrubs, distributed throughout the world and predominantly found in the Mediterranean basin [1,2]. They are widely used as spices, herbal tea, and insecticide in addition to flavor and fragrance materials. Among these, *Thymus quinquecostatus* (Bak-ri-hyang) is a scrubby subshrub and an important aromatic plant in Korea. Two varieties of *T. quinquecostatus* such as *T. quinquecostatus* Celak and *T. quinquecostatus* var. *japonica* are found in Korea [3]. In traditional systems of medicine, *T. quinquecostatus* is used for the treatment of cough, inflammation, preventing excessive intestinal gas, and diaphoresis [3,4,5]. Recent scientific studies reported that *T. quinquecostatus* has antioxidant, antimicrobial, insecticidal, immunological, antidiabetic, and antitumor properties [6,7,8]. The essential oil of *T. quinquecostatus* is extensively used in cosmetic industries for fragrance purposes. Owing to its medicinal and aromatic properties, it is also broadly used in pharmaceutical and food industries [9,10,11]. The most abundant components in *T. quinquecostatus* essential oil are thymol, γ-terpinene, and *p*-cymene [8].

Currently, thyme seeds are commercially available in the market. However, most thyme cultivars are not yet chemically or genetically characterized. In particular, *T. quinquecostatus* cultivar is often mixed with other thyme cultivars during cultivation and in nature. Therefore, it is important to validate the methods for the identification of the Korean native thyme cultivar, *T. quinquecostatus*. The essential oil components were commonly used to examine variations between populations [12,13,14]. It was reported that the genetic constitution and environmental conditions highly influenced the yield and essential oil composition of various plant species [1]. The chemical composition of essential oils might be further altered due to cross hybridization, morphogenesis, polyploidization, extraction methods, drying conditions, stages of harvesting, etc. According to the type, the major components represent 60–95% of the total essential oil. The essential oil composition might vary with the cultivar type. In *T. vulgaris*, seven different chemotypes, such as thymol, carvacrol, geraniol, linalool, thujanol-4, terpinen-4-ol, and 1,8-cineole are described [15,16]. However, the identification of the factors responsible for the chemical polymorphism registered within species is the most challenging aspect of the essential oil analysis.

In addition, the morphological similarity and anatomical features of thyme cultivars create a problem for the correct identification. The genetic variation of plants was also affected by evolution in both inter- and intra-species. Since the distinctness of *Thymus* species from another is always challenging to identify, several characters might need to be considered. DNA-based molecular markers were used for the successful detection of this genetic variation, in the process of evolution, gene flow, and population diversity in many plant species. In recent decades, a number of molecular techniques were used to assess genetic diversity in plants. Among them, the PCR-based random amplified polymorphic DNAs (RAPD) were used for the identification of cultivar and genetic relationships, among and within plant species. Similarity banding pattern was scored for calculation of genetic relatedness [13]. Furthermore, RAPD analysis did not need any previous knowledge regarding the target sequence on the genome of the species [15]. When compared with other molecular markers, RAPD markers can produce a high percentage of polymorphism in plants with very similar genetic characteristics. RAPD analysis has various advantages, including easiness, rapidity, requiring a small amount of genomic DNA as a template, and the possibility for detecting dissimilarity in the coding and noncoding areas of the genome [17,18,19]. Previously, several authors have reported the use of RAPD markers to study the genetic diversity, phylogenetic relationship, and its combination with the analysis of essential oil composition in various *Thymus* species [1,13,15,20,21]. RAPD data are also important to solve the taxonomic issues within and among plant species. Furthermore, the physiological and morphological variations, essential oil composition, ploidy level, and the relationship between the chemical and genetic evaluations of the *Thymus* species were assessed [15].

In recent times, several companies have commercialized thyme seeds. Hence, Korean native thyme, *T. quinquecostatus* (Bak-ri-hyang) is always mingled with commercial thyme species during cultivation and marketing. In addition, there are no studies on the RAPD evaluation of *T. quinquecostatus* and most studies focus on its essential oil composition. In this context, the present study aimed to evaluate the chemical and genetic variations among *T. quinquecostatus* cultivars from Korea and six commercial *T. vulgaris* cultivars, by using different RAPD markers and essential oil profiles, in order to distinguish Korean native thyme, Bak-ri-hyang from commercial thyme cultivars.

## 2. Methods

### 2.1. Plant Materials

**Figure 1:**
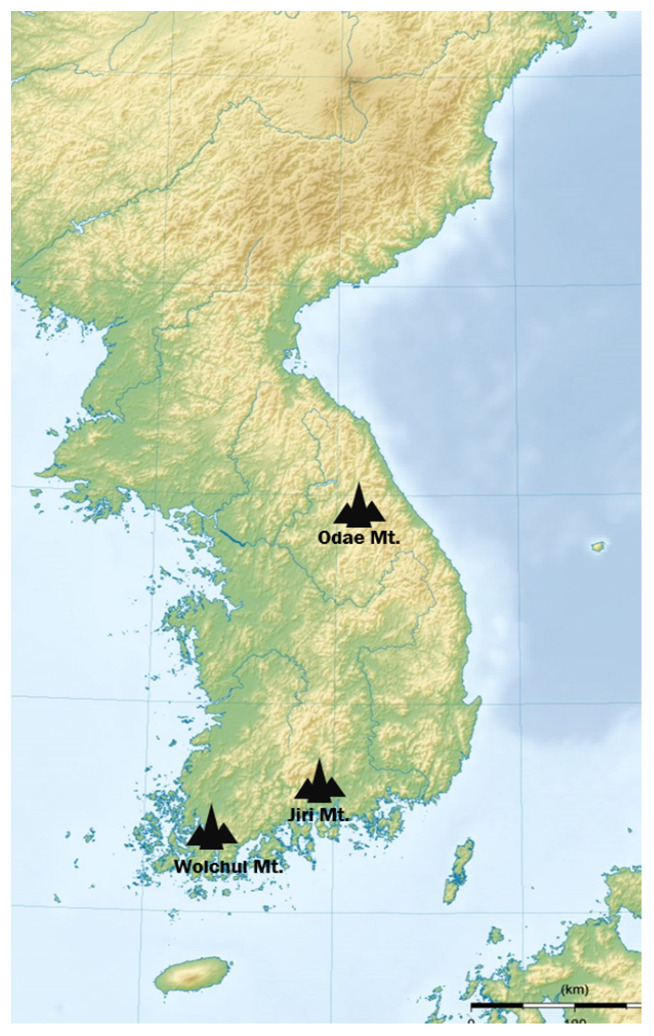
The map showing the collection sites of three accessions (Wolchul, Odae, and Jiri mountains) of *Thymus quinquecostatus* in South Korea.

**Figure 2:**
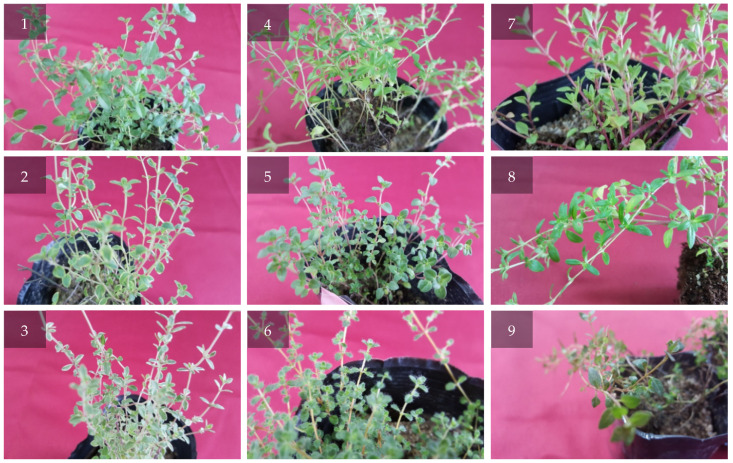
The morphology of six commercial *Thymus vulgaris* cultivars and three Korean native *Thymus quinquecostatus* cultivars. (1) Lemon; (2) golden; (3) carpet; (4) orange; (5) silver; (6) creeping; (7) Odae Mt.; (8) Wolchul Mt.; and (9) Jiri Mt.

First, a survey of the native thyme species (*T. quinquecostatus*) grown in Korea was carried out. We also interviewed experts who had ethnobotanical knowledge on Korean native thyme. According to their ethnobotanical information, we collected *T. quinquecostatus* from three accessions, such as Odae Mt, Wolchul Mt, and Jiri Mt in Korea, during April 2018 (Figure 1 and Figure 2). In addition, fresh plants of six *T. vulgaris* cultivars (lemon, golden lemon (golden), carpet, orange, silver, and creeping) were purchased from Daerim Horticulture, Gwachon, Happy Horticulture, Goyang and Nature Horticulture, Yangju, Republic of Korea (Figure 2).

The plants were authenticated and deposited in the Herbarium, Daejin University, Pocheon, Gyeonggi-do, Republic of Korea, with voucher numbers: lemon—DJU20180713, golden—DJU20180712, carpet—DJU20180717, orange—DJU20180715, silver—DJU20180714, creeping—DJU20180716, Odae Mt.—DJU20180718, Wolchul Mt.—DJU20180719, and Jiri Mt.—DJU20180720. The collected samples were kept at –20 °C for the essential oil analysis and –80 °C for the molecular analysis.

### 2.2. Morphological Characteristics

The morphological parameters such as stem type, stem branch, stem color, leaf shape, number of auxiliary leaves, and trichome position were observed for the six commercial and three Korean native thyme cultivars.

### 2.3. Essential Oil Extraction

The essential oil from nine thyme samples was isolated by steam distillation, using a Clevenger-type apparatus. The steam distillation was performed at 100 °C for 90 min. The essential oil isolation was carried out in triplicates and the yield (%) was calculated as volume (mL) of the isolated oil per 100 g of the fresh plant material. The isolated essential oil was dried using anhydrous sodium sulfate and stored at 4 °C, until tested. The color of essential oils obtained from the three Korean native *T. quinquecostatus* cultivars was measured, using the Chromameter CT-300 (Mintola Camera Co. Ltd., Japan). The intensity of the color was expressed in terms of L* lightness, a* greenness, and b* yellowness. The color values of L*, a*, and b* were taken in triplicates for each sample.

### 2.4. Gas Chromatography–Mass Spectrometry (GC–MS) Analysis

The identification of the essential oil components from different thyme cultivars was performed using a Varian CP3800 gas chromatograph coupled with a Varian 1200 L mass detector (Varian, CA, USA). The GC–MS was equipped with a VF-5MS polydimethylsiloxane capillary column (30 m × 0.25 mm × 0.25 μm). The oven temperature was programmed from 50 °C to 250 °C, at a rate of 5 °C/min. The injector temperature was 250 °C and the ionization detector temperature was 200 °C. Helium was the carrier gas (1 mL/min) and the injected volume of the sample was 2 μL, with a split ratio of 10:1. For mass spectra, an electron ionization system with ionization energy of 70 eV was used. The mass range was 50–500 m/z. The determination of the percentage composition of each component was based on the normalization of the GC peak areas. The identification of the essential oil components was based on the comparison of their retention indices (RIs), relative to a homologous series of *n*-alkanes (C_8_–C_22_) and mass spectra from the National Institute of Standards and Technology (NIST, 3.0) library and literature data [22].

### 2.5. DNA Extraction

The total genomic DNA was isolated from one gram of young leaves of plants, according to the CTAB (cetyl trimethylammonium bromide) extraction method [15]. DNA pellets were dissolved in TE (Tris–EDTA) buffer and RNA was removed by digestion with DNase-free RNase A. The purified total DNA was quantified and its quality was verified using a spectrophotometer, and a diluted solution with the same concentration (10 ng/μL) was prepared by adding TE buffer and was stored at 4 °C.

### 2.6. Randomly Amplified Polymorphic DNA (RAPD) Analysis

A total of 16 primers (OPA-09, OPA-10, OPA-11, OPA-12, OPA-13, OPA-14, OPA-15, OPA-16, OPA-17, OPA-18, OPA-19, OPA-20, OPB-01, OPB-02, OPB-03, and OPB-04) were used for the RAPD analysis (Table 1). The selection of primers was based on high polymorphisms and good reproducibility of the fragments generated. RAPD amplification was performed in a volume of 25 µL containing 10 ng total DNA, 1× PCR buffer, 3.0 mM MgCl_2_, 200 µM deoxynucleotide triphosphates (dNTPs), 1 μM primer, 1 µg/mL (w/v) Bovine Serum Albumin (BSA), and 1 unit *Taq* DNA polymerase (Invitrogen). The amplification reactions were performed in a thermocycler and consisted of an initial 5 min denaturation step at 95 °C, followed by 40 cycles of 20 s at 95 °C, 40 s at 35 °C, and 60 s at 72 °C. A final extension of 5 min at 72 °C completed the amplification. The PCR products were separated in 1.2% agarose gels 1× TAE buffer (Tris–Acetate). The gels were stained with ethidium bromide, visualized with a UV transilluminator.

### 2.7. Statistical Analysis

To calculate RAPD polymorphism, the RAPD markers were scored for the presence (1) or absence (0) of amplified bands for 9 thyme cultivars. Genetic similarity was estimated using the Jaccard’s coefficients. Cluster analysis was performed using the unweighted pair group method with an arithmetic mean (UPGMA), and dendrograms were drawn using NTSYS software version 2.02.

## 3. Results

### 3.1. Morphological Characteristics of the Thyme Cultivars

The morphological characteristics of six commercial and three Korean native thyme cultivars are presented in Table 2. In these, all three *T. quinquecostatus* cultivars had a creeping type of stem. On the other hand, lemon, golden, orange, and silver cultivars possessed an erect stem type. The length of the stem branch varied among different cultivars. In the creeping stem type, the length of the stem branch ranged from 2 to 8 cm, whereas the length of the stem branch in the erect type ranged from 3 to 11 cm. Carpet cultivar possessed a higher number of stem branches than the other cultivars. The shape of the leaves was mainly oval, followed by oblanceolate. In the case of the Bak-ri-hyang cultivars, the Odae and Jiri cultivars had an oval shape of leaves. The leaf shape of the Wolchul cultivar was oblanceolate. Furthermore, the Bak-ri-hyang cultivars possessed a higher number of auxiliary leaves when compared with the commercial thyme cultivars. The trichome position was mainly observed at the leaf petiole. In the Wolchul cultivar, the trichome position was observed at the leaf margin.

### 3.2. The Chemical Composition of Essential Oils

The yield and chemical composition of essentials oils obtained from the nine thyme cultivars are presented in Table 3 and Table 4. The essential oil components and their concentration produced by thyme cultivars were very diverse. The essential oil yields ranged between 0.12% and 0.43% (v/w) for the *T. quinquecostatus* cultivars. The highest yield was obtained from the Odae cultivar (0.43%). For commercial cultivars of *T. vulgaris*, the essential oil yields ranged from 0.23% to 0.33%. In these, the highest yields were obtained from the lemon and silver cultivars (0.34% and 0.33%, respectively), and the lowest yield was obtained from the carpet cultivar (0.23%). The color profile of the essential oils obtained from the *T. quinquecostatus* cultivars was measured. The L* value of the essential oils of the Wolchul, Odae, and Jiri cultivars was 92.48, 92.54, and 92.39, respectively. Wolchul and Jiri cultivars possessed similar a* value (0.18). With regards to the b* value, the Wolchul cultivar showed the highest value (2.29) and the Odae cultivar showed the lowest value (1.89). The total number of components in the analyzed essential oils ranged between 32 (creeping cultivar) and 43 (lemon cultivar). In these nine samples, twelve compounds were detected in all essential oil samples and these oils were dominated by monoterpenes, accounting for 79.95–92.16% with 0.03–46.47% of monoterpene hydrocarbons and 43.86–88.46% of oxygenated monoterpenes. Whereas sesquiterpenes achieved 6.50–29.17% with 5.83–15.07% of sesquiterpene hydrocarbons and 0.32%–17.23% of oxygenated sesquiterpenes.

**Figure 3:**
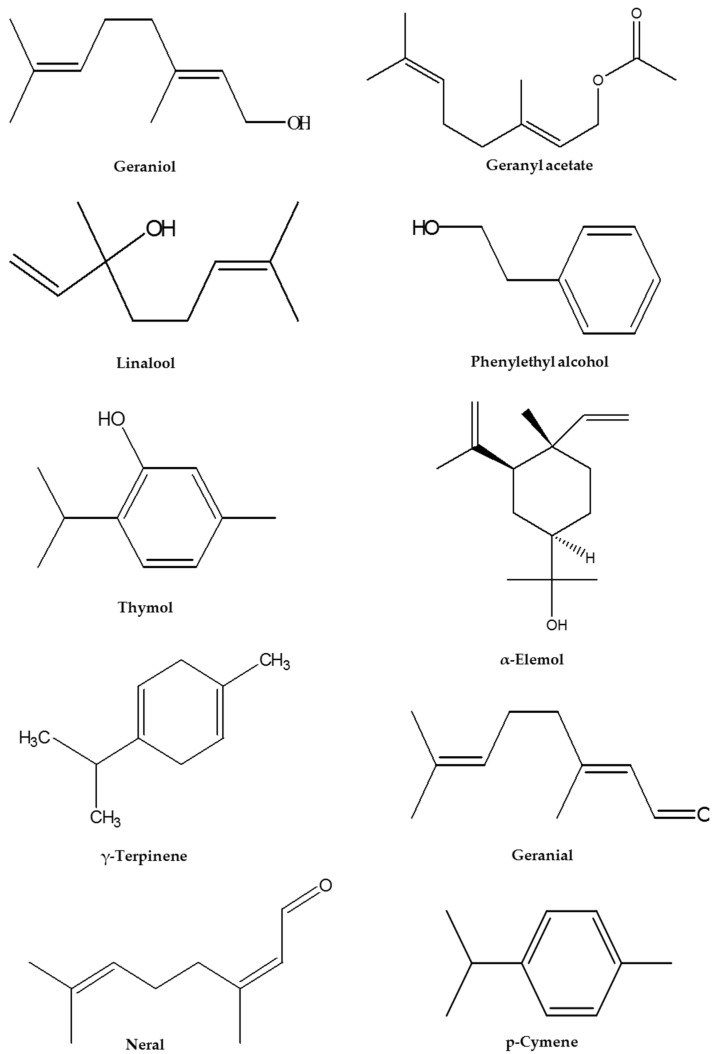
Structure of the major components identified in the essential oils of commercial *Thymus vulgaris* cultivars and Korean native *Thymus quinquecostatus* cultivars. Geraniol, geranyl acetate, linalool, phenylethyl alcohol, γ-terpinene, and thymol were identified as the major components, comprising >20% in at least one of the essential oil obtained from the different cultivars.

Geraniol, geranyl acetate, linalool, phenylethyl alcohol, γ-terpinene, and thymol were detected as the most abundant components, which comprised more than 20% in at least one essential oil (Figure 3). Results of the essential oil composition revealed that all three *T. quinquecostatus* cultivars tested belonged to different chemotypes (Supplementary Figure S1). Cultivars of Wolchul, Odae, and Jiri were mainly composed of geraniol (42.94%), thymol (30.54%), and linalool (47.89%), respectively. In the case of the commercial cultivars of *T. vulgaris*, the lemon and silver cultivars belonged to the thymol chemotype (43.91% and 66.24%, respectively). On the other hand, the creeping, golden, and orange cultivars belonged to the geraniol chemotype (29.57%, 65.99%, and 44.70%, respectively). With regards to the carpet cultivar, linalool (48.16%) was recorded as the most abundant component. Furthermore, geranyl acetate was detected as a major component in the Wolchul (26.49%) and silver (29.86%) cultivars. γ-Terpinene (23.92%) and *p*-cymene (11.13%) were major components in the Odae cultivar. In the creeping cultivar, neral (11.75%) and geranial (18.21%) were also recorded as major components. Other important compounds detected in all essential oils were caryophyllene (2.87%–7.02%), borneol (0.41%–5.91%), β-bisabolene (0.23%–3.86%), and 1-octen-3-ol (0.39%–3.61%). α-Elemol (11.62% and 8.52%, respectively) was also recorded as a major component in the carpet and creeping cultivars.

### 3.3. RAPD Analysis

**Figure 4:**
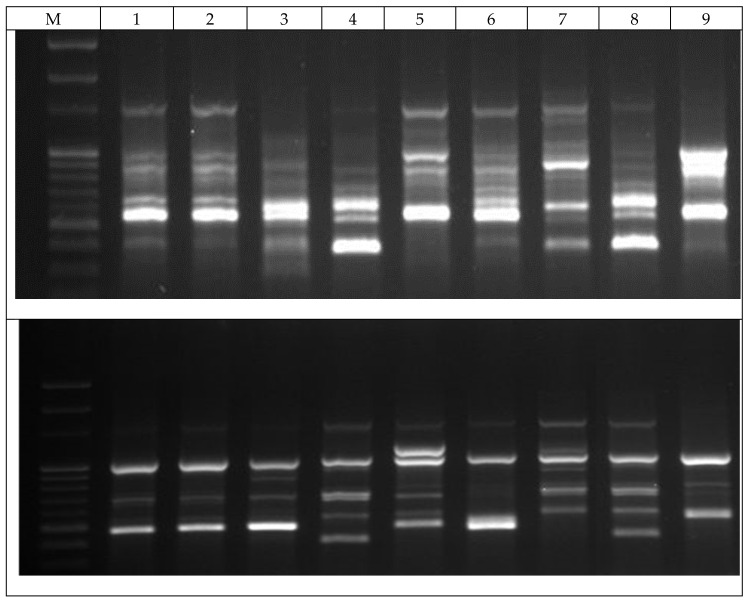
The example of a pattern among the three Korean native *Thymus quinquecostatus* cultivars and the six commercial *Thymus vulgaris* cultivars using OPB-01 (top) and OPA-11 (bottom) primers separated in 1.2% agarose gel electrophoresis. M, PCR marker; 1–6, commercial *Thymus vulgaris* cultivars: (1, lemon; 2, golden; 3, carpet; 4, orange; 5, silver; and 6, creeping); 7–9, Korean native *Thymus quinquecostatus* cultivars (7, Odae Mt; 8, Wolchul; 9, Jiri).

The molecular analysis revealed that the RAPD primers produced clear and reproducible polymorphic bands (Figure 4) among 9 thyme cultivars, and generated a total of 133 amplicons from 16 primers. The number of bands per primer varied from 4 (OPA-12, OPA-14, and OPB-04) to 16 (OPA-19), with an average of 8.31 bands per primer. In these, 124 amplicons were polymorphic, corresponding to 93.23% polymorphism (Table 5). Eight primers gave the highest percentage of polymorphism (100%), while the lowest percentage of polymorphism (75%) was obtained by OPA-12 and OPB-04 primers (Table 5).

**Figure 5:**
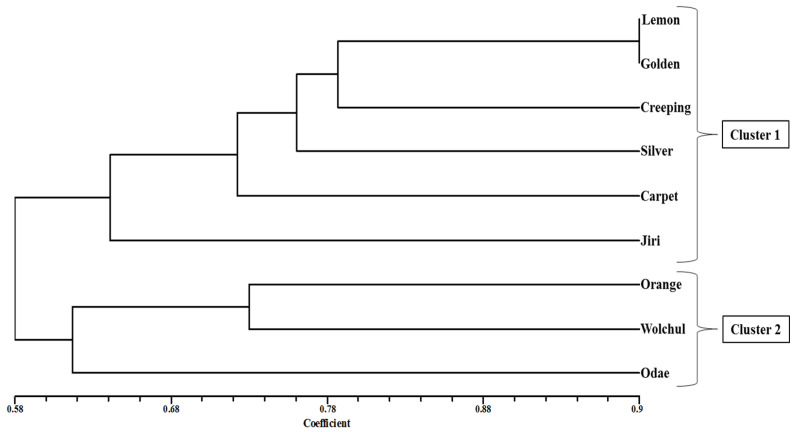
Clustering tree of the three *Thymus quinquecostatus* cultivars and the six commercial *Thymus vulgaris* cultivars, based on the unweighted pair-group method with the arithmetic average (UPGMA), using 16 RAPD markers.

The dendrogram realized from the RAPD markers grouped the 9 thyme cultivars into two major clusters and showed a clear separation (Figure 5). Levels of genetic similarity indices ranged from 0.58 to 0.98. Cluster 1 consisted of lemon, golden, creeping, silver, carpet, and Jiri. Whereas cluster 2 consisted of orange, Wolchul, and Odae.

## 4. Discussion

The identification of the *Thymus* species is extremely difficult because of the high levels of diversity within the genus. This genus contains several commercially important aromatic species. For this purpose, the relationship among the chemical composition of essential oils and molecular analysis was carried out for different *Thymus* species [20,23]. In this context, the essential oil composition and molecular analysis of nine thyme cultivars were investigated in this study, to distinguish between commercial thyme cultivars and Korean native thyme cultivars. In the morphological study, the *T. quinquecostatus* and *T. vulgaris* cultivars exhibited a significant level of variability in recorded parameters. In the qualitative traits, a considerable variability was observed in stem type, stem color, length and number of stem branches, leaf shape, and trichome position, among and within *T. quinquecostatus* and *T. vulgaris* cultivars.

The present study showed a high chemical diversity among nine thyme cultivars. Results revealed that essential oils from Korean cultivars (*T. quinquecostatus*) belonged to the geraniol, thymol, and linalool chemotypes. Essential oils from the commercial thyme cultivars (*T. vulgaris*) such as creeping, golden, and orange belonged to the geraniol chemotype and lemon, and the silver cultivars belonged to the thymol chemotype. Further, carpet cultivar belonged to the linalool chemotype. In particular, these essential oils were dominated by monoterpenes. 1-Octen-3-ol, γ-terpinene, linalool, borneol, α-terpineol, nerol, geraniol, thymol, β-cubebene, β-elemene, caryophyllene, β-bisabolene, butylated hydroxytoluene, β-sesquiphellandrene, and caryophyllene oxide were detected in all six essential oils from the commercial cultivars. With regards to the chemical composition of *T. vugaris* essential oils, seven different chemotypes such as thymol, carvacrol, linalool, geraniol, thujanol-4, terpineol, and 1,8-cineole were identified [15,16]. In the case of *T. quinquecostatus* essential oils, Shin and Kim [8] found that thymol (41.70%), γ-terpinene (16.00%), and *p*-cymene (13.00%) were the most prominent compounds. Similarly, thymol (30.54%), γ-terpinene (23.92%), and *p*-cymene (11.13%) were the major components in the essential oil obtained from the Odae cultivar. However, the major components in the essential oils obtained from Wolchul and Jiri cultivars of *T. quinquecostatus* were not identical. In the Wolchul cultivar, geraniol (42.94%) and geranyl acetate (26.49%) were detected as the major components, whereas linalool (47.89%) and thymol (15.98%) were found to be abundant in the Jiri cultivar.

Hudaib and Aburjai [24] determined variations in the composition of essential oils from cultivated and wild-growing plants of *T. vulgaris* grown in Jordan. Higher oil yields were obtained in plants growing wild, when compared to the cultivated plants. Among the four different samples, thymol (0.8–63.8%) and carvacrol (6.9–86.1%) were the most abundant components in the *T. vulgaris* essential oils. A study indicated that the essential oil composition of *T. vulgaris* highly varied both qualitatively and quantitatively during the vegetative cycle [25]. The variations in the yield and composition of essential oils could be influenced by various factors, such as the geographical region of the plant, plant’s maturity, cultivation practices, and weather parameters (temperature, humidity, sunlight duration, and rainfall) [26,27,28]. In addition, the genetic constitution of the cultivars also played a considerable role in the essential oil composition [1,25].

According to previous reports, it is difficult to distinguish *Thymus* species and cultivars by analyzing the essential oil profile alone. Hence, the combined analysis of chemical composition and molecular techniques was used for the correct identification of the different plant species. In recent decades, the correlation between the chemical composition and molecular analysis of different *Thymus* species were investigated by various researchers [1,13,20,21]. Previous studies showed that both essential oil composition and RAPD analysis could be used to distinguish different thyme cultivars, and especially, to determine their relationships [1]. In addition, RAPD analysis revealed high polymorphisms even when using closely related genotypes. Even though the essential oil composition of plants was different from one another, RAPD analysis clustered these plants together, owing to their similar genetic background [15].

In the present study, 16 primers were used to amplify segments of DNA of the genome of three Korean thyme cultivars and six commercial thyme cultivars, to investigate the genetic variations. A total of 133 bands were obtained and the average percentage of the polymorphic bands was 93.23%. Based on the RAPD data, the similarity of the cultivars, estimated by the Jaccard’s coefficient, is depicted in Figure 5. The nine cultivars of thyme fell into two clusters. Cluster 1 was formed by six cultivars (lemon, golden, creeping, silver, carpet, and Jiri) and cluster 2 by three cultivars (orange, Wolchul, and Odae). This emphasized the obvious variation between the Korean cultivars (except Jiri cultivar) and the commercial cultivars. The dendrogram indicated a clear separation of *T. quinquecostatus* from *T. vulgaris*, with the exception of the Jiri cultivar. According to the RAPD similarity matrix, it was observed that the Wolchul and Odae cultivars were closely related. Nevertheless, there was no significant relationship between the essential oil composition and RAPD data. The ability to discriminate all studied cultivars using RAPD bands indicated that RAPD analysis can provide a rapid and inexpensive technique to identify phenotypically similar thyme cultivars.

Based on previous reports, a high correlation between genetic and chemical relationships was attained in several plants. These data indicated that the composition of the essential oil is regulated by a number of genes that are extensively distributed throughout the plant genome [1,29,30]. Khalil et al. [31] used RAPD analysis to determine the genetic relationship between *T. vulgaris* populations collected in Syria. In their study, 13 individuals were analyzed using 27 primers, which generated 180 polymorphic bands from 198 bands. The authors found a significant correlation between *T. vulgaris* populations and their geographic areas. The present study also proved that the geographic distribution had a significant influence on genetic variation. Comparing the groups formed by the cluster analysis based on RAPD data (Figure 5) and chemotype, based on essential oil composition, we can observe that the groups formed in both cases were not identical.

In another study, the composition of essential oils and genetic relationships between six commercial cultivars of *T. vulgaris* were analyzed. A total of 104 were polymorphic RAPD bands (63.8%) were obtained using 15 primers. Among 15 primers, the highest percentage of polymorphism was obtained by the OPA-05 primer (90.9%). Similar to the essential oil composition, the six *T. vulgaris* cultivars fell into two major clusters, according to the RAPD patterns, with a correlation coefficient of −0.779 [1]. The chemical and genetic variations of 20 taxa from four Hungarian *Thymus* species (*T. glabrescens*, *T. pannonicus*, *T. praecox*, and *T. pulegioides*) were studied by Pluhár et al. [23]. In the molecular analysis, 114 polymorphic RAPD bands (80.8%) were obtained using 13 primers. The results revealed that partial correlation was found between the essential oil and RAPD analyses. The essential oil composition and genetic variation in six micropropagated genotypes (in vitro and in vivo) of *T. saturejoides* were investigated by Nordine et al. [32]. RAPD results and the essential oil composition grouped these six genotypes into three clusters exhibiting significant intraspecific chemical and genetic differences. Furthermore, a significant correlation was observed between RAPD and essential oil composition obtained from the in vitro genotypes.

Similar to our report, several studies also reported that the combined use of RAPD and essential oil analyses were not significantly correlated. For example, the genetic and chemical relationships among 31 individuals of *T. caespititius* collected from the islands of Pico, Sao Jorge, and Terceira (Azores) were determined. In the RAPD analysis, 187 polymorphic bands were obtained using 17 primers. However, there was no close relationship between the collection site, the essential oil composition, and RAPD analysis [15]. Rustaiee et al. [20] also studied the essential oil composition and genetic variability between some *Thymus* species such as *T. daenensis* (two populations), *T. fallax*, *T. fedtschenkoi*, *T. migricus*, and *T. vulgaris*, using GC-MS and RAPD. Although the RAPD markers allowed a perfect distinction among different *Thymus* species according to their characteristic genetic background, there was no identical clustering with the essential oil composition. In addition, Masi et al. [33] found that the essential oil compositions did not match with the results achieved from agronomic and genetic analyses in *Ocimum basilicum*. In another study, there was no correlation between RAPD and the essential oil obtained from the in vivo genotypes of *T. saturejoides* [32]. Based on the previous and present studies, marker-assisted RAPD technique had a high advantage for the assessment of the genetic differences of plant species without prior molecular knowledge.

Results of the present study revealed that there was a significant correlation between the genetic and geographic distances of the Korean thyme cultivars (Wolchul and Odae cultivars), compared to the commercial thyme cultivars. However, the chemical polymorphism of these thyme cultivars is not well-understood. Hence, other molecular techniques should be investigated in order to understand this question in *T. quinquecostatus* and other *Thymus* cultivars.

## 5. Conclusions

The present study emphasized that RAPD analysis allowed a perfect distinction between the Korean thyme cultivars (Wolchul and Odae) and commercial thyme cultivars, based on their unique genetic background. However, the chemical composition of the Wolchul and Odae cultivars was not identical. Furthermore, there was no significant relationship between the RAPD data and essential oil composition of both *T. quinquecostatus* and *T. vulgaris* cultivars. The chemical composition and molecular data obtained in this study delivered a good starting point for future investigations. It could be concluded that the RAPD markers proved to be an effective tool for discriminating different *Thymus* species. The sample collection must be done from different geographical regions in Korea to understand the genetic and chemical variability of the *T. quinquecostatus* cultivars.

## Figures and Tables

**Table 1:** The names and sequences of the primers used for random amplified polymorphic DNA (RAPD) analysis.

S. No.	Primer	Sequence
1	OPA-09	GGGTAACGCC
2	OPA-10	GTGATCGCAG
3	OPA-11	CAATCGCCGT
4	OPA-12	TCGGCGATAG
5	OPA-13	CAGCACCCAC
6	OPA-14	TCTGTGCTGG
7	OPA-15	TTCCGAACCC
8	OPA-16	AGCCAGCGAA
9	OPA-17	GACCGCTTGT
10	OPA-18	AGGTGACCGT
11	OPA-19	CAAACGTCGG
12	OPA-20	GTTGCGATCC
13	OPB-01	GTTTCGCTCC
14	OPB-02	TGATCCCTGG
15	OPB-03	CATCCCCCTG
16	OPB-04	GGACTGGAGT

**Table 2:** Morphological characteristics of six commercial *Thymus vulgaris* cultivars and three Korean native *Thymus quinquecostatus* cultivars.

*Thymus* Sp.	Variety	Stem Type	Stem Branch		Stem Color			Leaf Shape	Number of Auxiliary Leaves	Trichome Position		
			Length (cm)	Number	Purple	Pale Purple	Pale Green			Leaf Petiole	Leaf Margin	Leaf Surface
*Thymus vulgaris*	Lemon	Erect	>3	2	-	-	√	Oval	0	-	-	-
	Golden	Erect	0~8	2	-	√	√	Elliptical	2	√	-	-
	Carpet	Creeping	0~4	4	-	√	-	Oval	0	√	√	√
	Orange	Erect	0~11	0e	-	-	√	Oblanceolate /Oblong	0~2	√	-	-
	Silver	Erect	0~5	0	-	√	-	Oblong	0~2	-	-	-
	Creeping	Creeping	0~2	2	√	-	-	Oval	0~2	√	-	-
Bak-ri-hyang (*Thymus quinquecostatus*)	Odae Mt.	Creeping	>2	0	√	-	-	Oval	2~6	√	-	-
	Wolchul Mt.	Creeping	0~8	2	-	√	√	Oblanceolate	2~8	-	√	-
	Jiri Mt.	Creeping	>2	0	√	-	-	Oval	2~6	√	-	-

**Table 3:** The yield and color of the essential oils isolated from the six commercial *Thymus vulgaris* cultivars and the three Korean native *Thymus quinquecostatus* cultivars.

*Thymus* Sp.	Cultivar	Essential Oil Yield (%)	Essential Oil Color
*Thymus vulgaris*	Lemon	0.34 ± 0.013	Yellow
	Golden	0.29 ± 0.020	Yellow
	Carpet	0.23 ± 0.015	Pale yellow
	Orange	0.29 ± 0.033	Pale yellow
	Silver	0.33 ± 0.042	Yellow
	Creeping	0.24 ± 0.021	Yellow
*Thymus quinquecostatus* (Bakrihyang)	Odae Mt.	0.43 ± 0.067	Yellow
	Wolchul Mt.	0.28 ± 0.017	White
	Jiri Mt.	0.12 ± 0.014	Yellow

**Table 4:** The chemical composition of essential oils isolated from the six commercial *Thymus vulgaris* cultivars and the three Korean native *Thymus quinquecostatus* cultivars.

S. No.	Compound Name	RI Lit	RI Cal	Area (%)								
				Lemon	Golden	Carpet	Orange	Silver	Creeping	Odae	Wolchul	Jiri
1	Tricyclene	926	923	-	-	-	-	-	-	0.05 ± 0.01	0.01 ± 0.00	-
2	α-Thujene	930	926	0.17 ± 0.01	-	-	-	0.04 ± 0.00	-	1.91 ± 0.50	0.01 ± 0.00	0.11 ± 0.02
3	α-Pinene	939	934	0.09 ± 0.01	-	-	-	0.02 ± 0.00	-	1.32 ± 0.32	0.26 ± 0.01	0.09 ± 0.02
4	Camphene	954	950	0.11 ± 0.01	-	-	0.01 ± 0.00	0.02 ± 0.00	-	1.69 ± 0.42	0.67 ± 0.01	0.18 ± 0.03
5	β-Pinene	974	978	0.24 ± 0.33	-	-	-	0.19 ± 0.02	-	1.89 ± 0.47	0.17 ± 0.01	0.30 ± 0.03
6	1-Octen-3-ol	979	985	3.61 ± 0.04	0.70 ± 0.06	0.57 ± 0.06	0.39 ± 0.02	1.46 ± 0.05	0.44 ± 0.03	2.81 ± 0.54	2.47 ± 0.02	3.29 ± 0.30
7	3-Octanone	983	989	0.76 ± 0.03	0.34 ± 0.02	-	0.01 ± 0.00	0.17 ± 0.01	0.0 8 ± 0.00	0.07 ± 0.00	0.82 ± 0.02	1.27 ± 0.20
8	3-Octanol	991	1000	0.38 ± 0.01	0.36 ± 0.02	-	0.06 ±0.01	0.23 ±0.02	0.15 ± 0.01	0.08 ± 0.02	0.70 ± 0.01	1.21 ± 0.19
9	α-Phellandrene	1002	1007	0.11 ± 0.01	-	-		0.04 ± 0.00		0.31 ± 0.09	-	0.05 ± 0.01
10	3-Carene	1011	1009	0.02 ± 0.01	-	-				0.08 ± 0.02	-	0.02 ± 0.01
11	*p*-Cymene	1024	1026	2.84 ± 0.10	0.41 ± 0.03	-	0.01 ± 0.00	2.22 ± 0.04		11.13 ± 0.62	0.05 ± 0.00	2.44 ± 0.15
12	D-Limonene	1029	1031	0.17 ± 0.01	0.01 ± 0.00	-		0.03 ± 0.01		0.53 ± 0.05	0.11 ± 0.01	0.10 ± 0.02
13	Eucalyptol	1032	1034	0.29 ± 0.01	0.09 ± 0.01	-	0.02 ± 0.01	0.41 ± 0.03		0.02 ± 0.01	0.44 ± 0.01	-
14	β-Ocimene	1050	1049	0.25 ± 0.01	-	-				0.09 ± 0.00	0.04 ± 0.00	0.12 ± 0.02
15	γ-Terpinene	1059	1060	8.44 ± 0.01	0.88 ± 0.06	0.05 ± 0.00	0.04 ± 0.00	3.85 ± 0.04	0.03 ± 0.00	23.92 ± 3.30	0.12 ± 0.00	3.43 ± 0.24
16	Sabinene hydrate	1070	1073	0.87 ± 0.03	0.05 ±0.01	-	0.15 ± 0.01	0.74 ± 0.06		3.02 ± 0.51	-	-
17	1-Nonen-3-ol	1078	1083	-	-	-	0.18 ± 0.01	0.30 ± 0.02	-	-	-	-
18	Terpinolene	1088	1087	0.93 ± 0.04	0.06 ± 0.01	-	-	-	-	3.55 ± 0.87	0.88 ± 0.02	0.56 ± 0.05
19	Nonanone	1090	1088	-	-	-				-	0.09 ± 0.01	-
20	Linalool	1096	1103	2.60 ± 0.12	0.47 ± 0.04	48.16 ± 0.67	0.36 ± 0.02	2.08 ± 0.11	3.86 ± 0.09	0.11 ± 0.03	1.49 ± 0.01	47.89 ± 3.11
21	Nonanal	1100	1108	-	-	-	-	-	-	-	0.07 ± 0.00	-
22	1-Octen-3-yl-acetate	1112	1109	-	-	-	-	-	-	-	0.02 ± 0.00	-
23	Chrysanthemal	1124	1120	-	-	-	-	-	0.43 ± 0.01	-	-	-
24	Verbenol	1141	1145	-	0.27 ± 0.01	-	-	-	0.85 ± 0.17	-	0.03 ± 0.00	-
25	Camphor	1146	1151	0.23 ± 0.03	-	0.04 ± 0.01	0.03 ± 0.00	0.62 ± 0.04	-	2.47 ± 0.45	-	0.10 ± 0.01
26	β-Pinene oxide	1159	1166	0.14 ± 0.02	0.02 ± 0.01	-	-	-	0.09 ± 0.02	-	-	-
27	Borneol	1169	1180	2.07 ± 0.07	0.41 ± 0.03	1.57 ± 0.16	3.05 ± 0.21	1.11 ± 0.11	0.73 ± 0.02	2.17 ± 0.49	5.91 ± 0.03	2.17 ± 0.34
28	Terpinen-4-ol	1177	1185	0.40 ± 0.01	-	0.15 ± 0.03	0.17 ± 0.01	0.37 ± 0.05	0.05 ± 0.00	1.49 ± 0.33	0.63 ± 0.03	0.17 ± 0.01
29	α-Terpineol	1188	1200	0.11 ± 0.03	0.05 ± 0.00	0.67 ± 0.01	0.04 ± 0.00	0.17 ± 0.04	0.03 ± 0.02	0.21 ± 0.06	0.09 ± 0.01	0.05 ± 0.01
30	Dihydrocarvone	1192	1202	-	-	-	0.04 ± 0.01	0.06 ± 0.02	-	-	0.13 ± 0.01	0.02 ± 0.01
31	Decanal	1201	1211	0.05 ± 0.01	-	0.06 ± 0.02	0.02 ± 0.00	-	-	0.07 ± 0.03	0.10 ± 0.01	0.05 ± 0.02
32	Nerol	1229	1233	2.66 ± 0.12	1.16 ± 0.05	0.17 ± 0.02	0.47 ± 0.03	1.99 ± 0.05	4.70 ± 0.05	-	1.34 ± 0.06	-
33	Thymol methyl ether	1235	1235	3.00 ± 0.09	0.26 ± 0.03	0.09 ± 0.03	-	2.17 ± 0.10	-	-	-	2.27 ± 0.16
34	Neral	1238	1243	0.99 ± 0.06	3.39 ± 0.21	-	0.26 ± 0.02	0.18 ± 0.05	11.75 ± 0.12	-	0.85 ± 0.03	-
35	2-Isopropyl-4-methylanisole	1244	1242	-	-	-	-	-	-	-	-	1.27 ± 0.18
36	Geraniol	1252	1262	6.03 ± 0.14	65.99 ± 2.30	0.73 ± 0.47	44.70 ± 0.67	3.02 ± 0.26	29.57 ± 0.65	0.35 ± 0.12	42.94 ± 0.32	0.03 ± 0.01
37	Geranial	1276	1264	1.49 ± 0.02	5.42 ± 0.18	0.03 ± 0.01	-	0.31 ± 0.04	18.21 ± 0.83	-	-	-
38	Cyclodecane	1271	1281	-	-	-	-	-	-	-	-	0.08 ± 0.03
39	1-Decanol	1269	1283	-	-	-	-	-	-	-	0.18 ± 0.01	-
40	Bornyl acetate	1285	1292	-	-	0.09 ± 0.00	0.19 ± 0.01	-	0.04 ± 0.01	-	0.53 ± 0.02	-
41	Thymol	1290	1295	43.91 ± 1.64	2.70 ± 0.09	13.36 ± 0.31	8.05 ± 0.03	66.24 ± 1.66	2.17 ± 0.08	30.54 ± 4.37	0.44 ± 0.02	15.98 ± 0.18
42	Carvacrol	1299	1305	-	-	0.78 ± 0.01	-	2.62 ± 0.03	-	-	-	0.27 ± 0.00
43	Methyl geranate	1324	1324	-	0.11 ± 0.01	-	-	-	0.03 ± 0.00	-	-	-
44	Thymol acetate	1352	1349	0.25 ± 0.01	-	-	0.57 ± 0.03	0.42 ± 0.03	-	0.29 ± 0.08	-	0.10 ± 0.02
45	Geranyl acetate	1381	1386	2.34 ± 0.11	2.89 ± 0.22	-	29.86 ± 0.84	1.09 ± 0.09	6.75 ± 0.08	0.20 ± 0.08	26.49 ± 0.05	-
46	β-Bourbonene	1388	1387	0.04 ± 0.00	0.13 ± 0.02	0.10 ± 0.01	-	-	0.15 ± 0.01	-	-	0.14 ± 0.02
47	β-Cubebene	1388	1390	2.61 ± 0.14	2.20 ± 0.17	5.07 ± 0.02	0.40 ± 0.04	0.81 ± 0.10	2.44 ± 0.16	0.19 ± 0.08	-	3.47 ± 0.27
48	β-Elemene	1390	1394	0.06 ± 0.00	0.07 ± 0.01	0.19 ± 0.02	1.02 ± 0.09	0.08 ± 0.01	0.10 ± 0.02	0.65 ± 0.22	-	0.67 ± 0.06
49	Caryophyllene	1419	1428	3.41 ± 0.16	4.56 ± 0.18	2.62 ± 0.01	4.74 ± 0.66	4.48 ± 0.27	2.87 ± 0.09	4.74 ± 1.04	4.73 ± 0.02	7.02 ± 0.39
50	Neryl propionate	1432	1439	-	0.46 ± 0.22	-	-	-	1.34 ± 0.17	-	-	-
51	α-Bergamotene	1434	1438	-	-	-	-	-	-	-	0.04 ± 0.01	-
52	γ-Elemene	1436	1442	-	0.03 ± 0.01	-	-	-	-	-	-	-
53	Aromadendrene	1441	1438	-	-	0.12 ± 0.02	-	-	-	0.08 ± 0.02	-	-
54	α-Humulene	1454	1463	3.51 ± 0.15	-	0.17 ± 0.01	-	0.18 ± 0.02	-	-	0.66 ± 0.03	-
55	β-Farnesene	1456	1454	-	-	0.04 ± 0.00	0.02 ± 0.01	-	-	-	-	-
56	Germacrene D	1480	1487	-	-	-	-	-	-	-	0.53 ± 0.02	-
57	β-Selinene	1490	1494	-	-	0.03 ± 0.00	-	-	-	-	-	-
58	α-Farnesene	1505	1506	-	-	-	-	-	-	-	-	0.40 ± 0.07
59	β-Bisabolene	1505	1514	2.22 ± 0.10	3.70 ± 0.26	2.47 ± 0.31	3.84 ± 0.45	0.23 ± 0.03	1.81 ± 0.11	1.21 ± 0.34	3.86 ± 0.03	3.00 ± 0.27
60	γ-Cadinene	1513	1518	-	-	-	-	-	-	0.05 ± 0.01	-	-
61	Butylated hydroxytoluene	1515	1518	0.14 ± 0.02	0.14 ± 0.02	0.60 ± 0.09	0.06 ± 0.01	0.18 ± 0.01	0.24 ± 0.04	-	-	-
62	δ-Cadinene	1523	1522	-	-	0.11 ± 0.01	-	-	-	0.12 ± 0.03	-	-
63	β-Sesquiphellandrene	1522	1528	0.12 ± 0.01	0.06 ± 0.01	1.02 ± 0.12	0.08 ± 0.01	0.05 ± 0.01	0.15 ± 0.03	-	0.10 ± 0.01	0.36 ± 0.07
64	α-Elemol	1549	1554	0.31 ± 0.02	0.05 ± 0.01	11.62 ± 0.25	-	0.04 ± 0.01	8.52 ± 0.52	-	-	-
65	Geranyl butyrate	1562	1558	-	1.08 ± 0.10	-	-	-	0.53 ± 0.12	-	-	-
66	cis-3-Hexenyl benzoate	1566	1570	-	-	0.09 ± 0.01	-	-	-	-	-	-
67	Germacrene D-4-ol	1575	1583	-	-	-	-	-	-	0.89 ± 0.38	-	-
68	Spathulenol	1578	1584	-	-	-	0.04 ± 0.00	-	-	-	0.03 ± 0.01	0.06 ± 0.01
69	Caryophyllene oxide	1583	1590	0.08 ± 0.01	0.15 ± 0.01	0.15 ± 0.02	0.20 ± 0.01	0.32 ± 0.06	0.23 ± 0.04	0.22 ± 0.10	0.26 ± 0.01	0.26 ± 0.07
70	Humulene epoxide II	1608	1618	-	-	-	-	-	-	-	0.02 ± 0.00	-
71	γ-Eudesmol	1632	1639	-	-	1.94 ± 0.19	-	-	0.09 ± 0.03	-	-	-
72	T-Muurolol	1646	1651	-	-	-	-	-	-	0.03 ± 0.01	-	-
73	β-Eudesmol	1650	1663	-	-	2.53 ± 0.26	-	-	0.26 ± 0.07	-	-	-
74	α-Cadinol	1654	1664	0.04 ± 0.01	0.02 ± 0.01	-	0.02 ± 0.01	-	-	0.08 ± 0.03	-	-
75	α-Bisabolol	1685	1691	0.04 ± 0.01	-	-	0.01 ± 0.00	-	-	-	-	-
76	Benzyl benzoate	1760	1767	-	-	0.30 ± 0.03	-	-	-	-	-	-
77	1-Hexadecanol	1875	1884	-	-	-	-	0.12 ± 0.02	-	-	-	-
	**Total**			**98.15 ± 0.51**	**98.67 ± 0.36**	**95.71 ± 1.60**	**99.09 ± 0.30**	**98.66 ± 0.11**	**98.61 ± 0.35**	**98.60 ± 0.56**	**98.32 ± 0.11**	**99.00 ± 0.30**
	Monoterpene hydrocarbons			13.38	1.37	0.05	0.21	7.11	0.03	46.47	2.31	7.39
	Oxygenated monoterpenes			72.17	84.67	66.48	88.46	85.05	79.87	43.86	85.76	76.22
	Sesquiterpene hydrocarbons			11.98	10.74	11.94	10.09	5.83	7.51	7.04	9.93	15.07
	Oxygenated sesquiterpenes			0.62	1.89	17.23	0.33	0.67	11.20	1.23	0.32	0.32

RI Lit, comparison of retention indices with those reported in the literature (Adams, 2007). RI Cal, retention indices relative to n-alkanes (C_8_–C_22_) on the VF-5MS column. Values are the mean of the three replicate determinations ± standard deviation.

**Table 5:** Bands and polymorphism revealed by the RAPD primers among the 9 *Thymus* cultivars.

Primer	Total No. of Bands	No. of Polymorphic Bands	Polymorphism (%)
OPA-09	10	10	100
OPA-10	8	8	100
OPA-11	12	11	91.67
OPA-12	4	3	75
OPA-13	10	8	80
OPA-14	4	4	100
OPA-15	8	7	87.50
OPA-16	10	9	90
OPA-17	5	5	100
OPA-18	9	9	100
OPA-19	16	16	100
OPA-20	10	9	90
OPB-01	8	8	100
OPB-02	7	7	100
OPB-03	8	7	87.5
OPB-04	4	3	75
**Total**	**133**	**124**	
**Average**	**8.31**	**7.75**	**93.23**

## Supplementary Materials

The following are available online at https://www.mdpi.com/2079-6382/9/6/289/s1, Figure S1: GC-MS chromatograms of essential oils from three Korean native *Thymus quinquecostatus* cultivars. A, Odae cultivar; B, Wolchul cultivar; C, Jiri cultivar.
