# Supplementary material for: Morphological, Chemical, and Genetic Characteristics of Korean Native Thyme Bak-Ri-Hyang (Thymus quinquecostatus Celak.)
Source: Antibiotics (Basel). 2020 May 28;9(6):289. doi: 10.3390/antibiotics9060289 (PMC7344789; doi:10.3390/antibiotics9060289)
Supplement: Supplementary file 1 [file antibiotics-09-00289-s001.pdf]

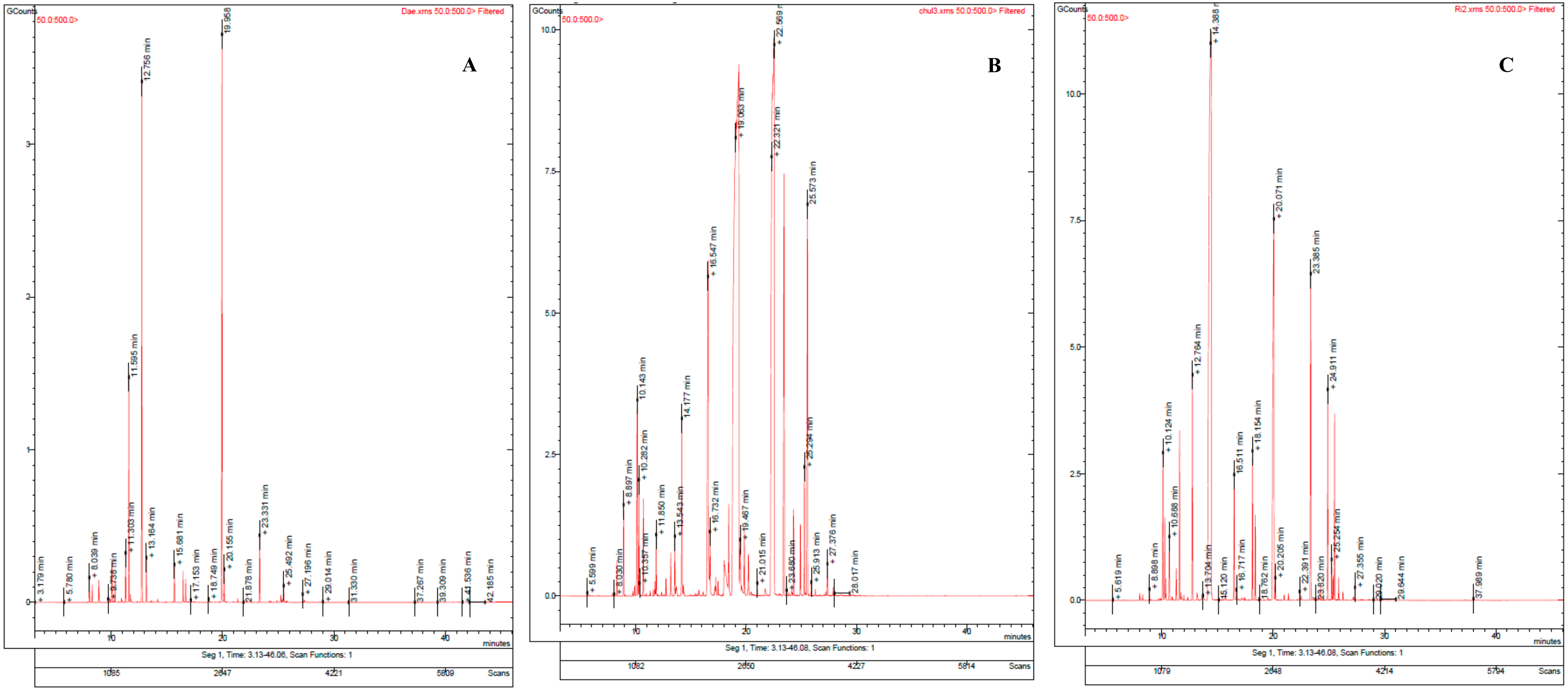

Supplementary Figure 1. GC-MS chromatograms of essential oils from three Korean native *Thymus quinquecostatus* cultivars. A, Odae cultivar; B, Wolchul cultivar; C, Jiri cultivar.
